# Comprehensive metabolomic profiling of osteosarcoma based on UHPLC-HRMS

**DOI:** 10.1007/s11306-020-01745-4

**Published:** 2020-11-18

**Authors:** Dongming Lv, Yutong Zou, Ziliang Zeng, Hao Yao, Shirong Ding, Yiying Bian, Lili Wen, Xianbiao Xie

**Affiliations:** 1 Department of Musculoskeletal Oncology, The First Affiliated Hospital of Sun Yat-sen University, Guangzhou, 510080 People’s Republic of China; 2 Guangdong Provincial Key Laboratory of Orthopedics and Traumatology, Guangzhou, 510080 People’s Republic of China; 3 Department of Anesthesiology, State Key Laboratory of Oncology in South China, Collaborative Innovation Center for Cancer Medicine, Sun Yat-sen University Cancer Center, Guangzhou, 510060 People’s Republic of China; 4 State Key Laboratory of Oncology in South China, Collaborative Innovation Center for Cancer Medicine, Guangdong Key Laboratory of Nasopharyngeal Carcinoma Diagnosis and Therapy, Sun Yat-sen University Cancer Center, Guangzhou, 510060 People’s Republic of China

**Keywords:** Metabolomics, Osteosarcoma, LC-MS, Biomarker

## Abstract

**Introduction:**

Osteosarcoma (OS) is the most common primary malignant bone tumor in children and adolescents. An increasing number of studies have demonstrated that tumor proliferation and metastasis are closely related to complex metabolic reprogramming. However, there are limited data to provide a comprehensive metabolic picture of osteosarcoma.

**Objectives:**

Our study aims to identify aberrant metabolic pathways and seek potential adjuvant biomarkers for osteosarcoma.

**Methods:**

Serum samples were collected from 65 osteosarcoma patients and 30 healthy controls. Nontargeted metabolomic profiling was performed by liquid chromatography-mass spectrometry (LC-MS) based on univariate and multivariate statistical analyses.

**Results:**

The OPLS-DA model analysis identified clear separations among groups. We identified a set of differential metabolites such as higher serum levels of adenosine-5-monophosphate, inosine-5-monophosphate and guanosine monophosphate in primary OS patients compared to healthy controls, and higher serum levels of 5-aminopentanamide, 13(S)-HpOTrE (FA 18:3 + 2O) and methionine sulfoxide in lung metastatic OS patients compared to primary OS patients, revealing aberrant metabolic features during the proliferation and metastasis of osteosarcoma. We found a group of metabolites especially lactic acid and glutamic acid, with AUC values of 0.97 and 0.98, which could serve as potential adjuvant diagnostic biomarkers for primary osteosarcoma, and a panel of 2 metabolites, 5-aminopentanamide and 13(S)-HpOTrE (FA 18:3 + 2O), with an AUC value of 0.92, that had good monitoring ability for lung metastases.

**Conclusions:**

Our study provides new insight into the aberrant metabolic features of osteosarcoma. The potential biomarkers identified here may have translational significance.

**Electronic supplementary material:**

The online version of this article (doi:10.1007/s11306-020-01745-4) contains supplementary material, which is available to authorized users.

## Introduction

Osteosarcoma is the most common primary malignant bone tumor in children and adolescents, mainly occurring between the ages of 10 and 25 years (Isakoff et al. 2015). With the introduction of neoadjuvant chemotherapy and advances in surgical limb salvage techniques, the 5 year overall survival rate for osteosarcoma is approximately 60% (Isakoff et al. 2015; Gianferante et al. 2017). Osteosarcoma has a strong tendency to cause lung metastasis, which may commonly lead to a poor prognosis. Over the past three decades, the clinical treatment of osteosarcoma has remained at a “plateau”. Oncologists have tried to improve the overall prognosis of OS patients by increasing the dose of chemotherapy drugs and ministering different combinations of chemotherapy, but the effect has not been satisfactory(Eaton et al. 2020). Thus, it is urgent to understand the development of osteosarcoma more comprehensively at the molecular level.

There is increasing evidence that cancer cells show significant metabolic disorders compared with normal cells. Cancer development and progression can be regulated by the energy transition from fermentable metabolites to respiratory metabolites (Seyfried et al. 2014). Metabolic reprogramming controlled by a complex network of coupled enzymatic reactions favors the development of cancers (Yoo et al. 2020; Zhang et al. 2019). A previous study showed that the Warburg effect or aerobic glycolysis represents an adaptive phenomenon that is commonly observed in brain tumors, which is closely related to stem cell differentiation, drug resistance and tumor recurrence (Venneti and Thompson 2017). Reprogramming of metabolic pathways including the tricarboxylic acid cycle, oxygen sensing and the aberrant metabolism of fatty acids, glucose, glutamine and arginine enables clear cell renal cell carcinoma cells to rapidly proliferate and survive under stress conditions or escape the immune response (Wettersten et al. 2017). Metabolomics, through measuring metabolite concentrations, provides possibilities for understanding the comprehensive metabolic status of biological systems associated with diseases (Jang et al. 2018). A growing number of studies have utilized this technique to search for biomarkers associated with tumor status to improve clinical prediction and diagnosis (Ferrarini et al. 2019; Battini et al. 2017; Jin et al. 2014). However, there are limited data to provide a comprehensive metabolic picture of osteosarcoma.

In this study, we collected serum samples from 65 osteosarcoma patients and 30 healthy controls. A serum metabolic profile based on lipid chromatography-mass spectrometry was generated to identify the aberrant metabolic pathway in osteosarcoma, and seek potential adjuvant biomarkers that could improve the diagnosis and monitoring ability for osteosarcoma.

## Materials and methods

### Study population

Ninety-five participants were included throughout the study, 30 of whom were healthy and 65 of whom were tumor patients. Sixty-five patients with a histologic diagnosis of osteosarcoma were enrolled from the First Affiliated Hospital of Sun Yat-sen University between January 2016 and July 2018. Among them, 32 patients were diagnosed with osteosarcoma without metastasis and the rest had lung metastasis. According to Enneking staging, the nonmetastatic OS patients were in stage II, including 18 in stage IIA and 14 in stage IIB, and the lung metastatic OS patients were all in stage III. All cohort patients provided written informed consent. The study was approved by the ethics committee of the First Affiliated Hospital of Sun Yat-sen University and the approval number is [2020]277. Detailed clinical information about the patients is presented in Table 1.

The inclusion criteria were as follows: (1) patients who were diagnosed with histologically confirmed conventional high-grade osteosarcoma by at least 2 orthopedists and 2 pathologists; (2) patients who underwent initial diagnosis and received a standard treatment protocol at our center; (3) patients who did not receive any antitumor therapy before admission to our center; (4) patients who underwent chest computed tomography (CT) to confirm the presence or absence of lung metastasis; and (5) patients for whom complete clinical and follow-up data were available.

**Table 1:** Clinical Information of osteosarcoma patients and healthy controls

	Healthy controls	Nonmetastatic OS patients	Lung metastatic OS patients
No. of subjects	30	32	33
Age, years, mean ± SD	22 ± 1.55	15.28 ± 6.67	19.07 ± 10.99
Gender (male/female)	16/14	16/16	21/12
Lesion location			
Humerus		3	2
Femur		17	16
Tibia		10	12
Fibula		2	1
Rib		0	1
Ilium		0	1
ALP (U/L)		222.5 ± 220.3	349.7 ± 494.3
LDH (U/L)		260.1 ± 109.1	328.7 ± 225.0
Enneking staging			
I		0	0
II		IIA: 18; IIB: 14	0
III		0	33

*OS* osteosarcoma, *ALP* alkaline phosphatase, *LDH* lactic dehydrogenase

## Sample preparation

Serum samples were collected before or after lung metastasis and frozen at − 80 °C until the beginning of sample preparation. Fifty microliters of thawed serum and 200 µL of cold methanol were combined, vortexed for 60 s, and kept at − 20 °C overnight. Following centrifugation at 18,000×*g* and 4 °C for 15 min, 200 µL of the supernatant was evaporated to dryness under a nitrogen stream. The residue was redissolved in 100 µL of 50% acetonitrile (v/v). Quality control (QC) samples pooled from all samples were prepared and analyzed with the same procedure used for the experimental samples.

## Nontargeted metabolomics analysis with the UHPLC-HRMS platform

Chromatographic separation was performed on a Thermo Fisher Vanquish UHPLC system with a Waters BEH C18 column (2.1 mm × 100 mm, 1.7 µm). The eluents were analyzed on a ThermoFisher Q Exactive™ plus Hybrid Quadrupole-Orbitrap™ Mass Spectrometer (QE plus) in heated electrospray ionization positive (HESI+) and negative (HESI−) mode. The spray voltage was set to 3200 V for HESI + and 3000 V for HESI−. The capillary and probe heater temperatures were 320 °C and 350 °C, respectively. The sheath gas flow rate was 50 (Arb), and the aux gas flow rate was 15 (Arb). The S-lens RF level was 50 (Arb). The full scan was operated at a high resolution of 70,000 FWHM (m/z = 200) in the range of 70–1050 m/z with an AGC target setting of 1 × 10^6^. Simultaneously, the fragment ion information of the top 10 precursors in each scan was acquired by data-dependent acquisition (DDA) with the HCD energy at 15, 30 and 45 eV, a mass resolution of 17,500 FWHM, and an AGC target of 5 × 10^5^.

## Data processing and metabolite identification

The raw data from the UHPLC-Q Exactive plus system were first transformed to mzXML format by ProteoWizard and then processed by the XCMS and CAMERA packages in the R software platform. In the XCMS package, peak picking (method = centWave, ppm = 5, peakwidth = c(5,20), snthresh = 10), alignment (bw = 6 and 3 for the first and second grouping, respectively), and retention time correction (method = obiwarp) were conducted. In the CAMERA package, annotations of isotope peaks, adducts, and fragments were performed with default parameters. The final data were exported as a peak table file, including observations (sample name), variables (rt_mz), and peak areas. The data were normalized against total peak areas before univariate and multivariate statistics were performed.

The accurate m/z of precursors and product ions were matched against online databases including mzCloud, Metlin, HMDB, MassBank, and local LipidBlast, and the in-house standard library including retention time, accurate precursors, and product ions.

### Statistical analysis

For multivariate statistical analysis, the normalized data were imported to SIMCA software (version 14.1, Umetrics, Umeå, Sweden), where the data were preprocessed by Pareto scaling and mean centering before OPLS-DA was performed. The model quality is described by the R2X or R2Y and Q2 values.

For univariate statistical analysis, the normalized data were analyzed in the “muma” software package in the R platform, where a parametric test was performed on data with a normal distribution by Welch’s *t* test, while a nonparametric test was performed on data with an abnormal distribution by the Wilcoxon Mann-Whitney test. The Benjamin-Hochberg method was used to calculate the false discovery rate (FDR). Those with *p* values lower than 0.05 and FDR values lower than 0.2 were considered statistically significant. Metabolic pathway enrichment analysis was performed on the following website (https://www.metaboanalyst.ca/). The relative amount of each metabolite was plotted by GraphPad Prism 7. Hierarchical cluster (HCL) analysis was performed by the MultiExperiment Viewer (Mev, version 4.9.0, Dana-Farber Cancer Institute, MA, USA). The area under the receiver operating characteristic (ROC) curve of primary discriminators was calculated using SPSS software.

## Results

### LC/MS-based metabolomics screening among the healthy group and the osteosarcoma groups

**Fig. 1:**
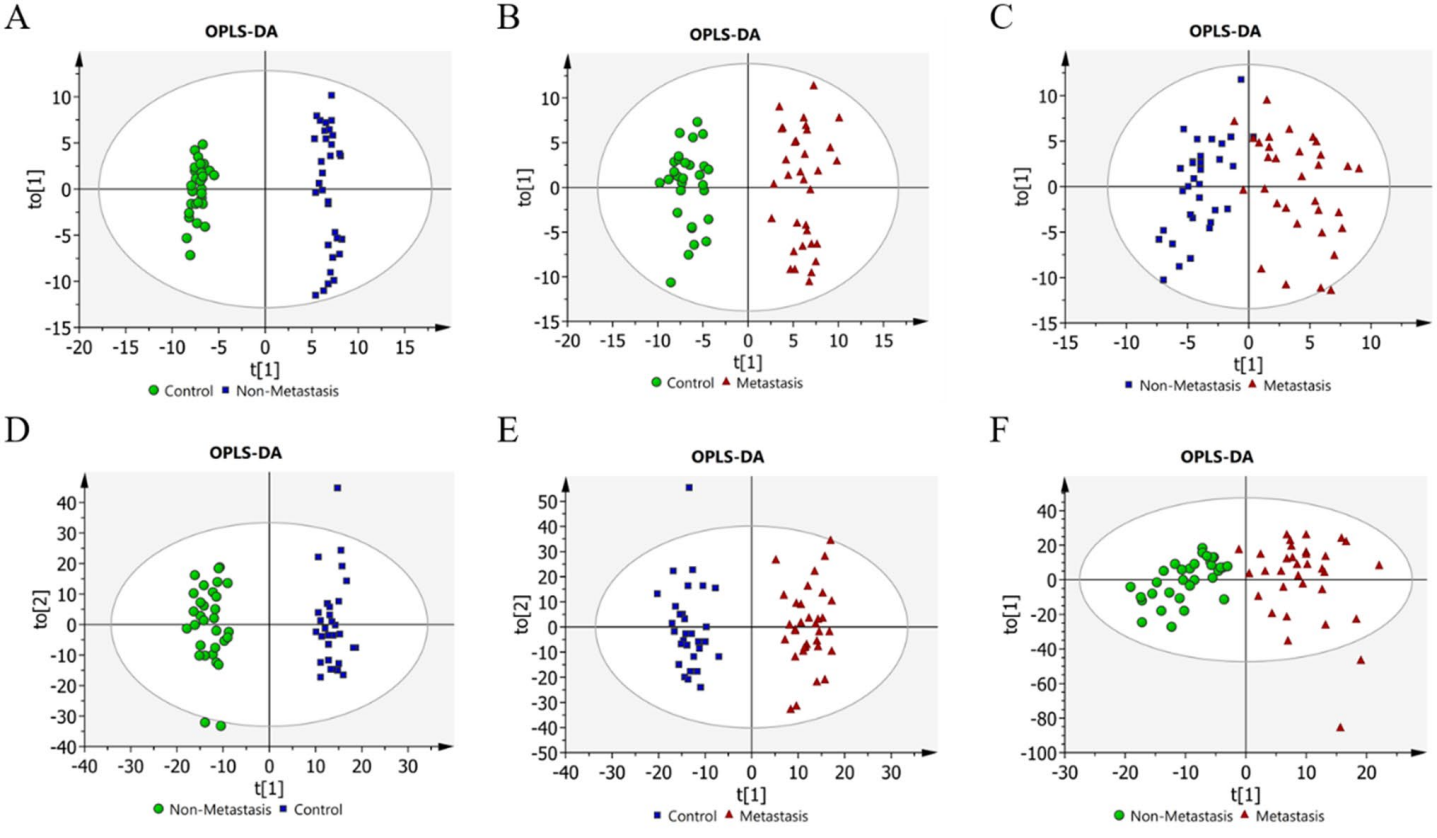
Score plot presenting the separation for pair splitting among samples through OPLS-DA. A-C OPLS-DA score plots for negative mode. Nonmetastasis vs. control, R^2^X = 0.291, R^2^Y = 0.989, Q^2^ = 0.91; Metastasis vs. control, R^2^X = 0.221, R^2^Y = 0.939, Q^2^ = 0.744; Metastasis vs. nonmetastasis, R^2^X = 0.139, R^2^Y = 0.751, Q^2^ = 0.0761. D-F OPLS-DA score plots for positive mode. Nonmetastasis vs. control, R^2^X = 0.19, R^2^Y = 0.971, Q^2^ = 0.678; Metastasis vs. control, R^2^X = 0.202, R^2^Y = 0.947, Q^2^ = 0.616; Metastasis vs. nonmetastasis, R^2^X = 0.14, R^2^Y = 0.789, Q^2^ = 0.0163

To reveal the different metabolic patterns during the development of osteosarcoma, LC/MS based metabolomics screening was conducted among the healthy group and the osteosarcoma groups. In both positive and negative ion modes, the OPLS-DA model can effectively distinguish among groups (Fig. 1a and f). Interestingly, we observed slight sample dispersion in the nonmetastatic group and lung metastatic group. This may be due to the different complications of tumor patients during the treatment process, and the corresponding treatment plan adjustments.

## Discovery of metabolic features for osteosarcoma

To identify the differential metabolites among groups, we obtained the variance importance (VIP) of all the ion peaks from ESI + and ESI− analysis modes. All identified metabolites and their detailed information were visible in the supplementary table.

In the comparison of nonmetastatic OS patients to healthy controls, using a combination of statistical significance and VIP values (> 1), we found 69 differential metabolic features, among which 31 metabolites were decreased and 38 metabolites were increased. According to the metabolic pathway analysis, the metabolic pathways that were significantly changed in nonmetastatic OS patients included alanine, aspartate and glutamate metabolism, phenylalanine, tyrosine and tryptophan biosynthesis, arginine biosynthesis, histidine metabolism, phenylalanine metabolism, and purine metabolism (Figure S1A).

In the comparison of metastatic OS patients to healthy controls, with the same criteria, 71 differential metabolites were identified, among which 32 metabolites were decreased and 39 metabolites were increased. The differential metabolites were mainly involved in arginine biosynthesis, histidine metabolism, alanine, aspartate and glutamate metabolism, and purine metabolism (Figure S1B).

**Fig. 2:**
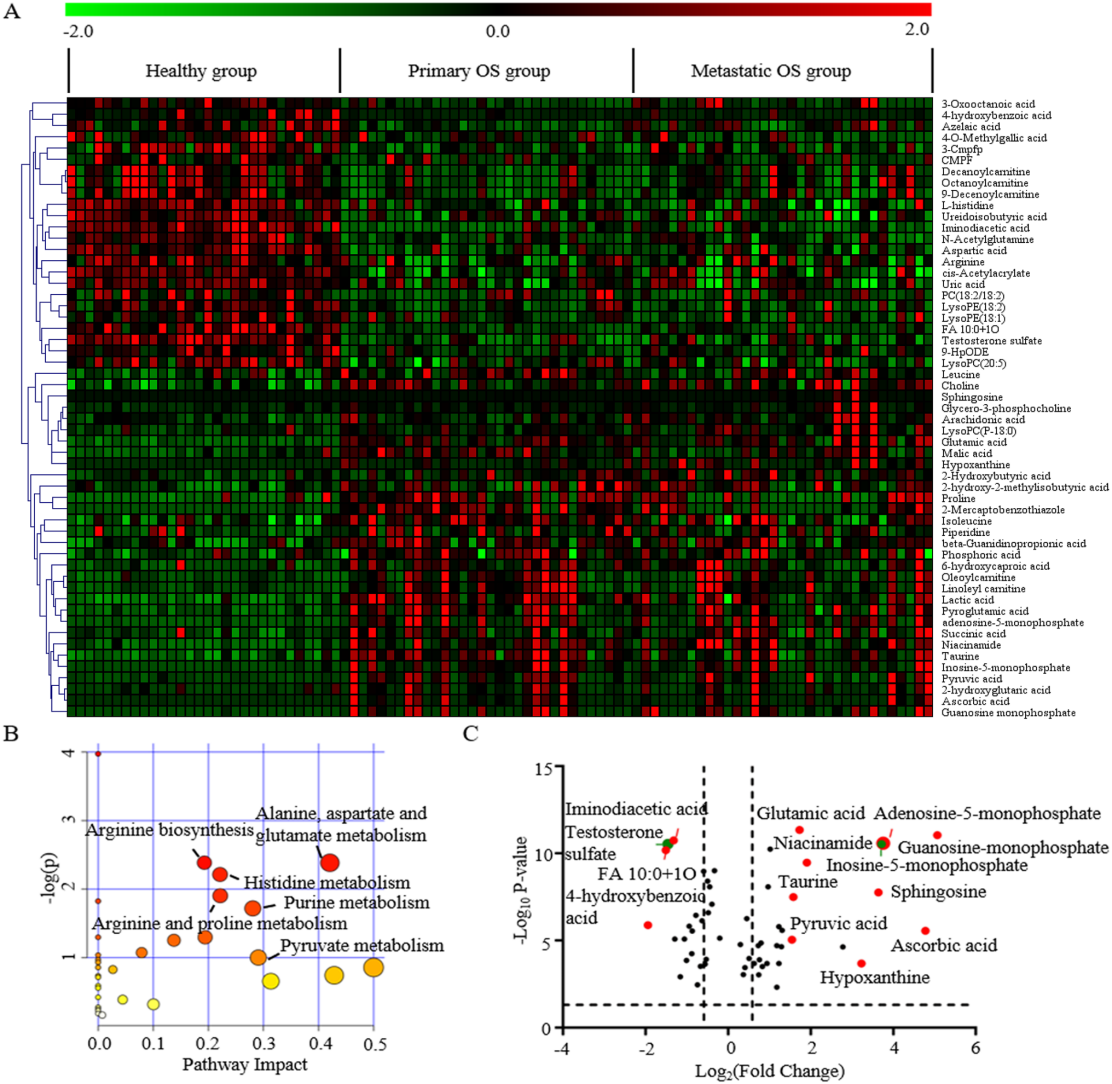
Identification of metabolic characteristics between osteosarcoma patients and healthy controls. A Heat map displaying the change direction of the discriminators. Upregulated metabolites are shown in red, while downregulated metabolites are shown green. B Pathway enrichment analysis of differential metabolites between OS patients and healthy controls. The abscissa represents the influencing factor of path topological analysis. The ordinate represents the *p* value of pathway enrichment analysis (negative logarithm). The larger the circle is, the larger the influence factor is. The deeper red color has the larger value of − log(*p*), which means the more significant enrichment. C A volcano plot shows the differential metabolites. Marked metabolites are the most distinct in OS patients compared with healthy controls

To better understand the perturbed metabolic pathways of osteosarcoma, we compared serum metabolites in OS patients (nonmetastasis + metastasis) and healthy controls and found that a total of 55 differential variables shared metabolic characteristics in OS patients with and without metastasis (Table 2). A heat map was generated to show the change direction of the discriminators (Fig. 2a). The KEGG pathway analysis showed that the perturbed metabolic pathways in OS patients included alanine, aspartate and glutamate metabolism, arginine biosynthesis, histidine metabolism, arginine and proline metabolism, purine metabolism, and pyruvate metabolism, which were commonly changed in many cancers (Fig. 2b).

**Table 2:** Differential serum metabolites with common metabolic characteristics in the nonmetastatic OS group and lung metastatic OS group

Metabolites	Nonmetastasis vs. control				Metastasis vs. control			
	*P* value^a^	FDR value^b^	VIP^c^	Log_2_FC^d^	*p* value^a^	FDR value^b^	VIP^c^	Log_2_FC^d^
4-Hydroxybenzoic acid	0.00	0.00	1.11	− 2.06	0.00	0.00	1.11	− 1.83
FA 10:0 + 1O	0.00	0.00	1.53	− 1.67	0.00	0.00	1.53	− 1.36
Testosterone sulfate	0.00	0.00	9.40	− 1.58	0.00	0.00	9.40	− 1.36
Iminodiacetic acid	0.00	0.00	2.25	− 1.37	0.00	0.00	2.25	− 1.27
3-Cmpfp(3-carboxy-4-methyl-5-pentyl-2-furanpropanoic acid)	0.00	0.00	3.55	− 1.52	0.00	0.00	3.55	− 1.10
CMPF(3-Carboxy-4-methyl-5-propyl-2-furanpropionic acid)	0.00	0.01	4.68	− 1.11	0.01	0.03	4.68	− 1.21
Decanoylcarnitine	0.00	0.00	2.56	− 1.35	0.00	0.01	1.94	− 0.83
4-O-Methylgallic acid	0.02	0.08	3.22	− 0.63	0.00	0.00	3.22	− 1.50
9-HpODE	0.00	0.00	2.20	− 1.14	0.00	0.00	2.20	− 0.76
Octanoylcarnitine	0.00	0.00	1.96	− 1.16	0.00	0.02	1.39	− 0.66
9-Decenoylcarnitine	0.00	0.00	1.94	− 1.09	0.00	0.00	1.53	− 0.69
LysoPE(18:2)	0.00	0.00	3.69	− 0.80	0.00	0.00	2.65	− 0.76
PC(18:2/18:2)	0.01	0.03	4.16	− 0.71	0.01	0.05	3.66	− 0.77
3-Oxooctanoic acid	0.00	0.00	1.64	− 1.26	0.00	0.01	1.64	− 0.20
LysoPE(18:1)	0.02	0.08	2.29	− 0.65	0.00	0.00	1.70	− 0.68
Cis-Acetylacrylate	0.00	0.00	1.40	− 0.70	0.00	0.00	1.40	− 0.50
Arginine	0.00	0.00	2.37	− 0.62	0.00	0.01	2.34	− 0.50
Azelaic acid	0.00	0.00	1.55	− 0.75	0.03	0.09	1.55	− 0.34
L-histidine	0.00	0.00	1.16	− 0.45	0.00	0.00	1.16	− 0.55
Ureidoisobutyric acid	0.00	0.00	2.25	− 0.44	0.00	0.00	2.25	− 0.52
N-Acetylglutamine	0.00	0.00	1.70	− 0.46	0.00	0.00	1.72	− 0.43
Uric acid	0.00	0.00	5.43	− 0.37	0.00	0.00	5.43	− 0.42
Aspartic acid	0.00	0.00	1.33	− 0.38	0.00	0.00	1.33	− 0.29
LysoPC(20:5)	0.00	0.00	2.71	− 0.19	0.00	0.00	2.75	− 0.20
Isoleucine	0.00	0.00	11.90	0.37	0.02	0.08	7.55	0.23
Piperidine	0.00	0.01	6.00	0.51	0.03	0.11	1.72	0.22
Leucine	0.00	0.00	11.49	0.52	0.01	0.05	6.22	0.30
Choline	0.00	0.00	5.63	0.39	0.00	0.00	6.45	0.51
Phosphoric acid	0.00	0.00	4.19	0.55	0.01	0.03	4.19	0.47
2-Hydroxybutyric acid	0.00	0.01	4.84	0.46	0.00	0.00	4.84	0.78
Proline	0.00	0.00	1.51	0.88	0.00	0.02	1.02	0.57
Succinic acid	0.00	0.00	2.82	0.77	0.02	0.07	2.82	0.72
2-hydroxy-2-methylisobutyric acid	0.00	0.01	1.53	0.78	0.00	0.00	1.53	0.74
Arachidonic acid	0.03	0.09	3.94	0.49	0.00	0.00	3.94	1.09
6-Hydroxycaproic acid	0.00	0.00	1.70	0.80	0.01	0.02	1.70	0.79
Beta-Guanidinopropionic acid	0.00	0.00	7.51	0.96	0.01	0.05	6.98	0.93
Pyroglutamic acid	0.00	0.00	10.42	1.03	0.00	0.00	8.93	0.90
Lactic acid	0.00	0.00	28.95	1.17	0.00	0.00	28.95	0.85
LysoPC(P-18:0)	0.00	0.00	1.61	0.64	0.00	0.00	3.97	1.63
Oleoylcarnitine	0.00	0.00	1.83	1.20	0.00	0.00	1.83	1.15
2-Hydroxyglutaric acid	0.00	0.01	1.10	1.19	0.05	0.15	1.10	1.17
Glycero-3-phosphocholine	0.00	0.00	1.52	0.76	0.00	0.00	2.83	1.67
Malic acid	0.00	0.00	2.70	1.29	0.00	0.01	2.70	1.16
Linoleyl carnitine	0.00	0.00	1.72	1.41	0.00	0.00	1.48	1.20
Pyruvic acid	0.00	0.00	3.98	1.53	0.00	0.00	3.98	1.56
Taurine	0.00	0.00	2.10	1.78	0.00	0.00	2.10	1.36
Glutamic acid	0.00	0.00	3.04	1.61	0.00	0.00	2.93	1.84
Niacinamide	0.00	0.00	2.68	1.82	0.00	0.00	2.78	1.99
2-Mercaptobenzothiazole	0.00	0.00	1.79	3.08	0.03	0.10	1.79	2.41
Sphingosine	0.00	0.00	1.13	1.29	0.00	0.00	3.85	4.48
Hypoxanthine	0.00	0.00	1.11	2.29	0.00	0.00	1.80	3.79
Adenosine monophosphate	0.00	0.00	1.56	3.64	0.00	0.00	1.56	3.66
Inosine-5-monophosphate	0.00	0.00	1.66	3.89	0.00	0.00	1.66	3.54
Ascorbic acid	0.00	0.00	4.50	4.91	0.01	0.02	4.50	4.65
Guanosine monophosphate	0.00	0.00	1.18	5.09	0.00	0.00	1.15	5.05

^a^The *p* value was calculated using Student’s *t* test or nonparametric Mann-Whitney U test

^b^False discovery rate by Benjamin–Hochberg method

^c^Variable importance in the projection (VIP) was obtained in the OPLS-DA model

^d^Fold change was calculated as a binary logarithm between groups, where a positive value means that the average mass response of metabolites in the nonmetastasis/metastasis group is larger than that in the control group

Importantly, several intermediate metabolites (adenosine-5-monophosphate, AMP; inosine-5-monophosphate, IMP; guanosine monophosphate, GMP; and hypoxanthine) of the purine nucleotide de novo synthesis pathway were significantly increased in OS patients. If not considering the VIP value, the nucleic acid product xanthosine was also found to be increased. However, the end product of these metabolites, uric acid, was downregulated in OS patients, indicating that active purine metabolism was closely related to the development of osteosarcoma. Lactic acid, which had the greatest VIP value, was found to be significantly elevated in both nonmetastatic and metastatic OS groups. Other discriminating elevated products between OS patients and healthy controls, including ascorbic acid, niacinamide, taurine, glutamic acid and pyruvic acid, were also found. Additionally, serum 4-hydroxybenzoic acid, FA 10:0 + 1O, testosterone sulfate, iminodiacetic acid, 3-carboxy-4-methyl-5-propyl-2-furanpropionic acid (CMPF) and decanoylcarnitine were markedly downregulated in OS patients (Fig. 2c). These aberrant metabolites provide insight into the metabolic phenotype of osteosarcoma.

To explore metabolic changes during the progression of osteosarcoma, further investigation was performed to identify the potential changed metabolites between nonmetastatic OS patients and metastatic OS patients. According to the screening criteria above, we identified 9 metabolic products to discriminate nonmetastatic and metastatic OS patients. If considering only statistical differences, we could include 2 more metabolites for a better exploration of disease progression. Compared to the nonmetastatic OS patients, the metastatic patients showed marked increases in 5-aminopentanamide, 13(S)-HpOTrE(FA 18:3 + 2O), methionine sulfoxide and linoleic acid and obvious decreases in dehydroacetic acid, 4-O-methylgallic acid, glycoursodeoxycholic acid and sorbitol 6-phosphate.

## Determination of potential biomarkers for auxiliary diagnosis and monitoring

**Fig. 3:**
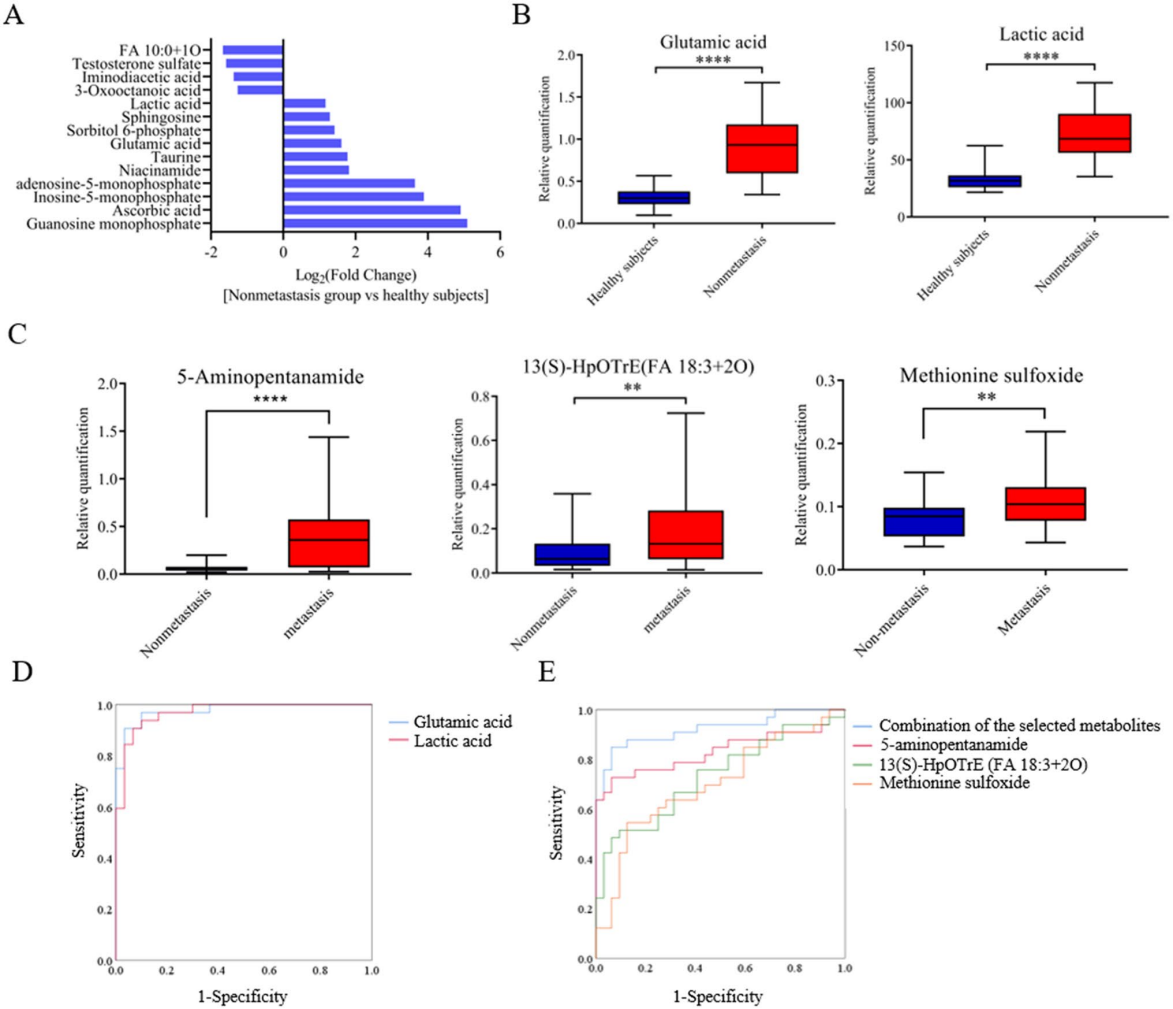
Determination of potential biomarkers for diagnosis and monitoring. A total of 14 metabolites with AUC values greater than 0.9. B Variations of glutamic acid and lactic acid between the nonmetastatic group and healthy controls. ****p < 0.0001. C Variations of 5-aminopentanamide, 13(S)-HpOTrE (FA 18:3 + 2O), and methionine sulfoxide between the metastatic group and the nonmetastatic group. **p < 0.0001, ****p < 0.0001. D ROC curves of glutamic acid and lactic acid providing an auxiliary diagnostic ability for osteosarcoma. AUC_glutamic acid_ = 0.98; AUC_lactic acid_ = 0.97. E ROC curves of the combination of the selected metabolites, 5-aminopentanamide, 13(S)-HpOTrE(FA 18:3 + 2O), and methionine sulfoxide providing monitoring ability for tumor metastasis. AUC_combination of the selected metabolites _= 0.92, AUC_5-aminopentanamide _= 0.83, AUC_13(S)−HpOTrE (FA 18:3+2O)_ = 0.73, AUC_methionine sulfoxide _= 0.70

To identify potential biomarkers for auxiliary diagnosis of osteosarcoma, further investigation based on a larger variation range was performed. Specifically, metabolites screened from the nonmetastatic OS groups and healthy controls, with appropriate fold changes (modulus > 2), VIP values (> 1) and statistical significance were eligible for candidates. To this end, we generated ROC curves for these selected candidates. There were 14 metabolites with AUC values greater than 0.9 (Fig. 3a). Of these, we were particularly interested in the two substances, glutamic acid and lactic acid (Fig. 3b), which had the greatest AUC values of 0.98 (0.95–1.00) and 0.97 (0.93–1.00) (Fig. 3d), respectively, indicating they could be potential auxiliary diagnostic markers.

The same approach was used to search for biomarkers for monitoring disease progression between the nonmetastatic and lung metastatic OS patients. We found 4 metabolites that met the criteria and ROC curves were generated individually for these 4 metabolites. There were 3 substances with AUC values greater than 0.7. These metabolites were 5-aminopentanamide, 13(S)-HpOTrE (FA 18:3 + 2O), and methionine sulfoxide (Fig. 3c). However, no differential metabolites with both high specificity and sensitivity were observed. It was necessary to combine multiple indicators to achieve better monitoring ability for disease progression. First, a binary logistic regression model was performed on the 4 differential metabolites and these metabolites were subjected to a stepwise variable selection method. In total, 2 metabolites were included in the equation, 5-aminopentanamide and 13(S)-HpOTrE (FA 18:3 + 2O). The prediction probability of the multi-index combination was then calculated. ROC analysis showed that this panel of biomarkers had an AUC value of 0.92 (0.85–0.99) (Fig. 3e), indicating that this combination could effectively discriminate metastatic OS patients among all OS patients.

## Discussion

Osteosarcoma is the most common primary malignant bone tumor in adolescents, with high rates of lung metastasis, disability and mortality. In this study, serum untargeted metabolomics analysis was performed on 65 patients with osteosarcoma and 30 healthy controls to reveal aberrant regulatory metabolic pathways in osteosarcoma. The OPLS-DA model analysis identified clear separations among groups. We found 55 differential variables shared metabolic characteristics in OS patients with or without metastasis compared to healthy controls, and they were mainly involved in alanine, aspartate and glutamate metabolism, arginine biosynthesis, histidine metabolism, arginine and proline metabolism, purine metabolism, and pyruvate metabolism. Among them, metabolites with the largest fold changes or VIP values, such as adenosine-5-monophosphate, inosine-5-monophosphate, guanosine monophosphate, pyroglutamic acid and lactic acid, have been reported to be closely related to malignancy of tumor in previous studies (Soares et al. 2015; Bahreyni et al. 2018; Ippolito et al. 2019). The global metabolomic profiling provides new insight into the aberrant metabolic features of osteosarcoma.

Based on the ROC curve analysis, we identified 14 metabolites with AUC values greater than 0.9 in the comparison of nonmetastatic OS patients and healthy controls (Fig. 3a). Of these metabolites, lactic acid and glutamic acid, with the greatest AUC values of 0.97 and 0.98, have a feasible auxiliary diagnostic ability of primary osteosarcoma. Both of the metabolites are tightly associated with the disorder of energy metabolism. Lactic acid, a metabolic product of aerobic glycolysis, was found to be elevated in the serum of primary osteosarcoma patients compared to healthy controls, and it had the largest VIP value (= 28.95) obtained from the OPLS-DA model. Previous study has found that lactic acid has no longer been viewed as a junk from the metabolism of fermenting cells. It could act as a powerful molecule that not only establish metabolic coupling between the tumor microenvironment(TME) and tumor cells but also transmit impacts on the cell signaling machinery(Ippolito et al. 2019). Lactic acid could act as an immunosuppressive metabolite to induce immune tolerance and promote tumor growth (Brand et al. 2016), and it is also frequently found at high levels in solid tumors (Romero-Garcia et al. 2016). Lactic acid produced by mesenchymal stem cells was reported to drive mitochondrial and oxidative phosphorylation as well as increase the migratory ability of osteosarcoma cells (Bonuccelli et al. 2014; Gorska-Ponikowska et al. 2017). Elevated serum LDH levels have been identified as prognostic indicators in patients with osteosarcoma (Gaetano Bacci 2004; Fu et al. 2018). The elevated serum lactic acid level we detected in OS patients may be the amplification effect of aberrant LDH expression in OS cells. Glutamic acid, a nonessential amino acid, could not only act as a bioenergetic substrate for cell proliferation but also as an excitatory neurotransmitter. It was found to be significantly elevated in the serum of OS patients in present study. A previous study demonstrated that osteosarcoma cells secrete glutamic acid and that blocking glutamic acid secretion with Riluzole could impair their proliferation (Liao et al. 2017). Compared to normal bone tissue, metabotropic glutamate receptor (mGluR) has been reported to be highly expressed in osteosarcoma tissues as well as related to poor prognosis (Yang et al. 2014), and it is well known that active mGluR signaling is linked to the PI3/AKT/mTOR pathway (Willard and Koochekpour 2013). Accordingly, the levels of serum lactic acid and glutamic acid could be probably utilized in the lab studies and could be performed as an auxiliary diagnostic biomarker for osteosarcoma at the time of initial diagnosis based on the imaging detection.

Currently, the 5-year overall survival rate of OS patients is approximately 60%, and one fourth of patients develop metastases at the initial diagnosis with lung metastases being the most common site. Timely discovery of lung metastases is particularly important for improving prognosis. Previous studies have reported that elevated levels of alkaline phosphatase (ALP) and lactate dehydrogenase (LDH) are tightly associated with lower event-free survival in patients with osteosarcoma (Hao et al. 2017; Fu et al. 2018). In our study, we analyzed serum levels of ALP and LDH in nonmetastatic and lung metastatic patients, and their AUC values were 0.50 and 0.57, respectively (Figure S2), which could not discriminate nonmetastatic and lung metastatic patients well. Consistently, we also noticed another previous study reporting that serum LDH and ALP levels differed significantly in patients with or without skeletal metastases but showed no difference in patients with or without lung metastases (Marais et al. 2015). To achieve monitoring ability for lung metastases, we identified a panel of 2 metabolites with an AUC value of 0.92, which could effectively discriminate lung metastatic OS patients among all OS patients. They were 5-aminopentanamide and 13(S)-HpOTrE (FA 18:3 + 2O). The α-linolenic acid (ALA) metabolite 13(S)-HpOTrE was reported to exhibit an anti-inflammatory effect mediated by induction of apoptosis and inhibition of autophagy in lipopolysaccharide-challenged macrophages (Kumar et al. 2016). Lysine was thought to be a nonspecific bridge molecule, that could associate antigen with T cells, causing T cells to produce a specific effect against the antigen. 5-aminopentanamide, as a product of the decarboxylation of lysine (Trisrivirat et al. 2020), may be related to the aberrant immune response in tumor patients. Our findings provide potential biomarkers for the timely discovery of lung metastases.

## Conclusions

The current metabolomics profiling using UHPLC-HRMS revealed a series of significantly different metabolites of osteosarcoma, which is consistent with the current research focus on cancer metabolism, providing us a new insight into the aberrant metabolic features of osteosarcoma. The potential adjuvant biomarkers for osteosarcoma were explored and ROC curves were generated. Serum levels of lactic acid and glutamic acid could serve as an auxiliary diagnostic indicator for primary osteosarcoma, and the combination of 5-aminopentanamide and 13(S)-HpOTrE (FA 18:3 + 2O) could be used as a monitoring indicator for lung metastases of osteosarcoma. Given the limited sample size, we will verify all these important findings in a larger osteosarcoma population in the future.

## Electronic supplementary material

Below is the link to the electronic supplementary material.

Electronic supplementary material 1 (RAR 298 kb)
